# A simple and unified protocol to purify all seven *Escherichia coli* RNA polymerase sigma factors

**DOI:** 10.1007/s13353-024-00870-3

**Published:** 2024-05-06

**Authors:** Barbara Kędzierska, Aleksandra Stodolna, Katarzyna Bryszkowska, Maciej Dylewski, Katarzyna Potrykus

**Affiliations:** Department of Bacterial Molecular Genetics, Faculty of Biology, University of Gdańsk, Gdańsk, Poland

**Keywords:** RNA polymerase, Sigma subunit, Sigma factor, Transcription, Protein purification, SUMO-tag

## Abstract

RNA polymerase sigma factors are indispensable in the process of bacterial transcription. They are responsible for a given gene’s promoter region recognition on template DNA and hence determine specificity of RNA polymerase and play a significant role in gene expression regulation. Here, we present a simple and unified protocol for purification of all seven *Escherichia coli* RNA polymerase sigma factors. In our approach, we took advantage of the His_8_-SUMO tag, known to increase protein solubilization. Sigma factors were first purified in N-terminal fusions with this tag, which was followed by tag removal with Ulp1 protease. This allowed to obtain proteins in their native form. In addition, the procedure is simple and requires only one resin type. With the general protocol we employed, we were able to successfully purify σ^D^, σ^E^, σ^S^, and σ^N^. Final step modification was required for σ^F^, while for σ^H^ and σ^FecI^, denaturing conditions had to be applied. All seven sigma factors were fully functional in forming an active holoenzyme with core RNA polymerase which we demonstrated with EMSA studies.

**Supplementary Information:**

The online version contains supplementary material available at 10.1007/s13353-024-00870-3.

## Introduction

In their natural environment, bacterial cells are exposed to constantly changing conditions to which they should react quickly in order to survive. This adaptation usually takes place at the step of gene expression regulation, allowing cells to change their metabolism according to conditions and resources available. Such regulation may involve transcriptional factors, second messengers (e.g., cAMP, (p)ppGpp), or simply RNA polymerase (RNAP) sigma factors.

Sigma factors are responsible for specificity of the RNAP holoenzyme, as they direct it towards gene promoter regions and allow the whole complex to bind the DNA and subsequently to initiate gene transcription. The core RNAP enzyme (i.e., without a sigma factor) is composed of two α subunits, and one of each, β, β’, and ω, and it does not bind DNA in a selective manner [for review see: Murakami and Darst 2003; Murakami 2015].

In *Escherichia coli*, there are seven sigma factors, where the major or “housekeeping” factor is σ^D^ (also called σ^70^; the numbers in sigma factor names come from approximate molecular weight of a given factor, in kDa). This factor is responsible for directing gene transcription under normal growth conditions. The other six sigma factors are often called “alternative” as they participate in gene expression regulation only under specific conditions [for review see: Österberg and del Peso-Santos 2011; Feklístov et al. 2014; Paget 2015; Davis et al. 2017; Helmann 2019]. For example, two sigma factors were found to be active under heat shock: σ^H^ (σ^32^) is already active under moderate heat shock conditions [Arsène et al. 2000], while σ^E^ (σ^24^) is active under extreme heat shock when denatured proteins accumulate in the periplasm or under conditions that cause periplasmic stress [Raina et al. 1995; Rouvière et al. 1995]. On the other hand, σ^F^ (σ^28^) controls expression of genes encoding flagella and those necessary for chemotaxis [Anderson et al. 2010], while σ^N^ (σ^54^) is active for example under nitrogen stress conditions [Shingler 2011]; yet σ^S^ (σ^38^) directs gene expression when cells are in the stationary phase of growth and is responsible for the general stress response [Battesti et al. 2011; Gottesman 2019]. The last sigma factor, σ^FecI^ (σ^19^) controls expression of Fe^3+^ transport genes [Van Hove et al. 1990; Angerer et al. 1995] and so far is the most obscure, as only one promoter has been identified to date to depend on this sigma factor [Angerer et al. 1995; Maeda et al. 2000; Shimada et al. 2017].

In order to study specific mechanisms governing transcription, in vitro methods are often employed. There, the reaction components must be very well defined, and the protein purity and proper folding is of the utmost importance. To date, many different approaches have been applied to purify *E. coli* sigma factors, very often involving purification from inclusion bodies (e.g., Angerer et al. 1995; Anthony et al. 2003; Enz et al. 2000; Maeda et al. 2000; Shikalov et al. 2019] and/or different resin types, such as those for ion exchange chromatography or size exclusion chromatography (e.g., Anthony et al. 2003; Liberek et al. 1992; Shikalov et al. 2019]. These protocols often require expensive resins or equipment that is not always readily available (e.g., MonoQ and the HPLC system). In addition, protein refolding from inclusion bodies may be problematic, as the proteins first aggregate in these dense structures and then are denatured under harsh conditions, followed by renaturation that may or may not be fully successful. On the other hand, there are protocols that involve the use of His-tag and immobilized metal affinity chromatography (IMAC), in combination with or without additional columns, where upon the final purification step the His-tag may or may not be removed by employing specific proteases, such as thrombin protease (in case of proteins expressed with the thrombin cleavage site) [Anthony et al. 2003; Lu et al. 2023; Obrist et al. 2009; Becker et al. 1999]. However, these protocols are not applicable to all *E. coli* sigma factors, while in case of studies comparing their activity, it would be ideal to purify all of them under the same conditions.

Here, we report on a simple protocol that can be employed for purification of all seven *E. coli* sigma factors. We took advantage of the SUMO tag, known to increase protein solubilization and decrease their aggregation [Marblestone et al. 2006]. In addition, such a tag was shown to enhance protein overexpression and decrease degradation when fused to the N-terminus of a given protein [Panavas et al. 2009; Peroutka Iii et al. 2011].

Indeed, the employed procedure allowed us to obtain all overproduced sigma factors in the soluble form, without the need for extraction from inclusion bodies. In addition, the SUMO tag was fused with His_8_ tag thanks to which all proteins could be purified by IMAC with the use of only one type of resin. Gravity flow chromatography that does not require any special equipment is employed. Upon His_8_-SUMO tag removal with specific SUMO protease, all sigma factors obtained are in their native form, i.e., without any additional tags. Finally, we demonstrate with the use of EMSA technique that the obtained factors are able to form functional RNAP holoenzymes.

## Materials and methods

### Bacterial strains and plasmids

Bacterial strains used in this study were *E. coli* MG1655 (ATTC #700926; used for sigma factor genes’ or their respective dependent promoter regions’ PCR amplification), *E. coli* DH5α (used for transformation with constructed plasmids and for plasmid maintenance), or *E. coli* BL21 (λDE3) (Novagen®; used for protein overexpression and purification). KP517 strain [Dylewski et al. 2019] was used for σ^70^ and σ^24^ - dependent promoter region amplification (this strain carries a modified *greA* promoter region that previously allowed us to study transcription by RNAP holoenzyme with the two mentioned sigma factors on the same DNA fragment).

**Fig. 1:**
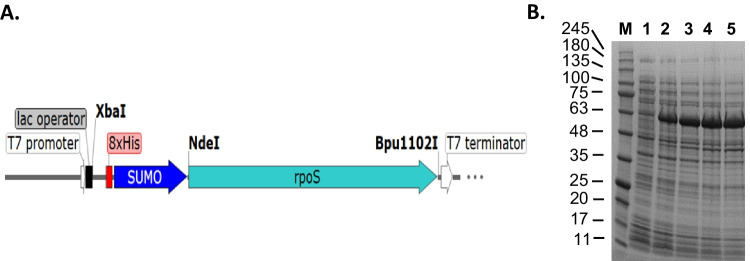
Overproduction of *E. coli* sigma factors. **A** Schematic representation of key elements in the plasmid used for sigma factor overproduction. As an example, plasmid used for σ^S^ overexpression is shown. SnapGene software was used. **B** Example of IPTG induction of sigma factor overproduction. σ^S^ overexpression is visualized by Coomassie blue-stained SDS-PAGE (10–20%). M, Perfect Tricolor protein ladder (Eurx); 1, uninduced cell lysate, 2–5, cell lysate obtained after 1, 2, 3, and 4-h induction with 1 mM IPTG. Equal amounts of lysate were loaded in each lane. Molecular weights of the marker bands are indicated on the left of the gel

All plasmids were constructed based on a pET28a derivative (pKB1, this work) in which His_8_-SUMO-tag has been inserted, followed by a convenient *Nde*I site. The genes of interest are cloned between *Nde*I and *Bpu*1102I sites, so the cloning/expression region’s structure is as follows: T7 promoter – *lac* operator – *Xba*I site – His_8_ – SUMO tag – *Nde*I site – gene of interest – *Bpu*1102I site – T7 terminator (original pET28a sequences are underlined) (Fig.1A). Sequence of the His_8_-SUMO tag has been derived from pCIOX (a gift from Dr. Andrea Mattevi; Addgene plasmid # 51300).

Plasmids used in this study are listed in Table S1, and primers used for PCR amplification of the sigma subunit genes are listed in Table S2. All DNA constructs obtained were verified by sequencing (Macrogen Europe). Constructed plasmids are available upon request.

### Protein overexpression and purification

In general, the protocol for sigma factor purification was as follows. The cells were grown at 32 °C in 250 ml of LB (supplemented with kanamycin, 30 μg/ml) until OD_600_~0.4, upon which protein overexpression was induced by addition of IPTG to 1 mM. The cultivation was continued for 3.5 h, after which the cells were harvested by centrifugation (3200 x g, 30 min, 4 °C) and stored at -20 °C until further use. The cell pellets were resuspended in 15 ml of ice-cold lysis buffer (20 mM Tris-Cl pH 8.0, 500 mM NaCl, 5% glycerol, 0.1 mM EDTA, 5 mM imidazole, 0.5 mM β-mercaptoethanol; LysB) supplemented with EDTA-free protease inhibitor cocktail (Thermo Fisher Scientific) and lysozyme (0.1 mg/ml) and incubated on ice for 30 min with gentle mixing every 10 min or so. Following sonication (on ice, 5 s on and off cycles, 2.5 min total sonication time; Vibra-Cell apparatus (Sonics)) and centrifugation (20,000 x g, 30 min, 4° C), the supernatants were applied on a BioRad disposable column pre-loaded with 2.5 ml of HisPur Ni-NTA resin (Thermo Fisher Scientific), pre-equilibrated with 15 ml of LysB. Resin was then washed twice with 30 ml of the wash buffer (same as LysB but containing 20 mM imidazole; WB-1), followed by His_8_-SUMO-tagged protein elution with three applications of 3 ml of the elution buffer (same as lysis buffer but with 250 mM imidazole; EB-1).

Fractions containing desired His-tagged protein (6–9 ml) were then pooled, placed in Slide-A-Lyzer cassettes (10 kDa cutoff; Thermo Fisher Scientific), and dialyzed overnight at 4°C in 900 ml of the following buffer: 20 mM Tris-HCl pH, 250 mM NaCl, and 5 % glycerol (DB-1); this was followed by dialysis in a fresh change of the same buffer for 1 h. The samples were removed and incubated with the in-house purified *S. cerevisiae* His-tagged Ulp1 SUMO protease (final concentration 10 μg/ml) [Sobala et al. 2019], incubated for 45 min at 4 °C on a rocker, and then applied on a BioRad disposable column pre-loaded with 1.5 ml of HisPur NiNTA resin, pre-equilibrated with 9 ml of DB-1. Flow-through fractions were collected, and the column was washed with 4 ml of the same buffer to elute the unbound proteins with removed His_8_-SUMO tag. These were then pooled and concentrated with the use of Amicon-15 Ultrafiltration devices (10 kDa molecular weight cut-off (MWCO)), and then the buffer was exchanged for 1 x TGED (20 mM Tris-Cl pH 8.0, 20% glycerol, 500 mM NaCl, 1 mM EDTA, 1 mM DTT) with the same devices. Finally, glycerol was added to 50%, and protein concentration was assessed with Qubit Protein Assay (Thermo Fisher Scientific).

All procedures were carried out at 4 °C with ice-cold buffers. At each step, the culture, cells and protein fractions were monitored for appropriate protein content by SDS-PAGE (10-20% Novex Tris-Glycine gels, Thermo Fisher Scientific) and Coomassie blue staining [Lawrence and Besir 2009].

For σ^F^, σ^FecI^, and σ^H^ purification, modified protocols were applied, as discussed in the Results and Discussion section. The detailed protocols are provided below.

For σ^F^ purification, the final protein prep in 1 x TGED was reapplied on a column pre-loaded with 1.5 ml of the HisPur NiNTA resin (pre-equilibrated with 9 ml of the 1x TGED buffer), and only the flow-through fraction was collected and used in further procedures.

For σ^FecI^, the first steps were the same as for other sigma factor purification, except that WB-1 contained 1 M NaCl. Next, denaturing buffers were applied. First, the resin was washed with 10 ml of 6 M guanidine hydrochloride, 20 mM imidazole, 20 mM Tris-HCl pH 8.0, and 500 mM NaCl (WB-2). Then, His-tagged proteins were eluted with a buffer the same as WB-2 but containing 250 mM imidazole (10 ml; EB-2), placed in Slyde-A-Lyzer cassettes, and dialyzed for 2 h against 1 L of the following buffer: 3 M urea, 20 mM Tris-Cl pH 8.0, 250 mM NaCl, and 5 % glycerol (DB-2). This was followed by 2 h dialysis against buffers containing decreasing urea concentrations (the same as DB-2 but containing 2 M and 1M urea (DB-3 and DB-4, respectively)), and finally overnight dialysis against DB-1. Next steps (His_8_-SUMO-tag cleavage, passing through a column, buffer exchange, and sample concentration) were the same as for the other sigma factors.

For σ^H^ purification, a modified wash procedure was followed by wash and dialysis under denaturing conditions. Upon applying cell lysate to the column, the resin was washed with 30 ml of WB-1supplemented with 5 mM ATP and 5 mM MgCl_2_ (WB-3), followed by 30 ml of WB-3 supplemented with 0.1 μg/ ml denatured proteins. Denatured proteins were obtained from 10 ml of *E. coli* MG1655 overnight culture (OD_600_~5.0, grown in LB); the cells were centrifuged (5,000 x g, 5 min), suspended in 2 ml of WB-1, sonicated (10 s on, 10 s off, 5 min total sonication time), centrifuged (14,000 x g, 10 min), incubated at 65 °C for 10 min, and re-centrifuged; protein concentration was estimated by A_280_ measurements (Nanodrop, Thermo). The resin was then washed with 30 ml of WB-3. The next steps were the same as for σ^FecI^ purification under denaturing conditions (10 ml wash with WB-2, and then elution (EB-2), dialysis against buffers containing decreasing urea concentrations (DB-2, DB-3, and DB-4), and finally against DB-1). Next steps (His_8_-SUMO-tag cleavage, passing through a column, buffer exchange and sample concentration) were the same as for the other sigma factors.

### Electrophoretic mobility shift assays (EMSA)

These assays were performed with PCR amplified, Cy5-end-labeled dsDNA linear templates containing promoter regions recognized by a given sigma factor. Primers used are listed in Table S3. And 20 μl reactions containing 30 nM *E. coli* RNA polymerase core enzyme (Epicentre) and 300 nM or 600 nM purified sigma factor (10 or 20 molar excess over the core, respectively) were assembled in a binding buffer (50 mM Tris-HCl pH 8.0, 50 mM KCl, 10 mM MgCl_2_, 10 mM β-mercaptoethanol, 2.5% glycerol) and incubated for 20 min at 37 °C to allow reconstitution of the holoenzyme. Then, Cy5-labeled DNA templates (10 nM, final) were added, and incubation was continued for another 15 min to allow DNA binding. Next, samples were loaded on 4% native polyacrylamide running gels (acrylamide/bisacrylamide ratio of 19:1) and electrophoresed in 0.5 x TBE buffer for 70 or 85 min (depending on the DNA length) at 100 V, room temperature. Detection of the Cy5–labeled DNA was performed using Typhoon scanner (GE Healthcare).

## Results and discussion

### Cloning of plasmids for overexpression of sigma factors

Appropriate sigma factor genes were cloned into a pET28a derivative containing His_8_-SUMO tag between *Nde*I and *Bpu*1102I restriction sites (see Materials and Methods for details and Fig. 1A). Gene expression was driven from a T7 RNA polymerase dependent promoter. DNA fragments for cloning were amplified from wild type *E. coli* chromosomal DNA with several exceptions (described below).

Since *rpoN* (σ^N^ encoding gene) contains a *Bpu*1102I site close to its 5’ terminus, an amplification primer was designed to introduce a silent mutation destroying that site. Similarly, *rpoH* (σ^H^ encoding gene) contains a *Bpu*1102I site at its 3’ terminus. In this case, a silent mutation was introduced by amplification of two separate fragments by PCR and then combining them into one fragment in a final PCR reaction. In case of *rpoD* (σ^D^ encoding gene), which contains both *Nde*I and *Bpu*1102I sites in its native sequence, a DNA fragment carrying silent mutations at these sites was obtained commercially (GeneArt service, Thermo Fisher Scientific; sequence provided in Supplementary Data) and used as template for subsequent PCR amplification and cloning. All DNA constructs obtained were verified by sequencing.

### Sigma factor purification: general protocol

The plasmids obtained were introduced into BL21(λDE3) strain carrying T7 RNAP gene under an IPTG inducible promoter. To verify expression, appropriate strains were first grown in 20 ml of LB at 32 °C to OD_600_~0.4 upon which IPTG was added to 1 mM and growth was continued for 4 h. Cell samples were removed at every hour, and protein content was monitored by SDS-PAGE. In most cases, overexpressed protein accumulation reached saturation at 3–4 h after induction (Fig. 1B and data not shown). Thus, for large-scale sigma factor overproduction, 3.5-h induction was used in general.

In our first attempts to purify *E. coli* RNAP sigma factors, we employed a standard procedure for His_8_-SUMO tagged proteins used in our laboratory, based on ion metal affinity chromatography (IMAC) (e.g., [Sobala et al. 2019]); see Materials and Methods for details). Briefly, the cells are grown in 250 ml of LB to OD_600_~0.4 at 32 °C, induced with IPTG, and then collected by centrifugation and frozen. The cell pellets are then resuspended in a lysis buffer, sonicated, and supernatants are applied to a column preloaded with Ni^2+^-NTA resin. Upon several washes with a buffer containing low concentration of imidazole (20 mM), the desired proteins are eluted with a buffer containing high imidazole concentration (250 mM), and dialyzed overnight in a buffer that allows for Ulp1 SUMO protease activity, which is used at a subsequent step to remove the His_8_-SUMO tag from purified sigma factors. Since the protease itself is also His-tagged, passing the whole sample through another Ni^2+^-NTA resin column allows for removal of both, the cleaved His_8_-SUMO tag, and the protease. In addition, if the prep is contaminated with proteins that interact with the resin but that were not removed at previous steps (i.e., they were not removed by washes of the first column), that second column allows for such clean-up. The flow-through sample is then collected, the column is washed with a small volume of the same buffer to elute unbound proteins present in the column dead volume, the pooled sample is concentrated, and the buffer is exchanged for sigma factor storage buffer (1 x TGED).

**Fig. 2:**
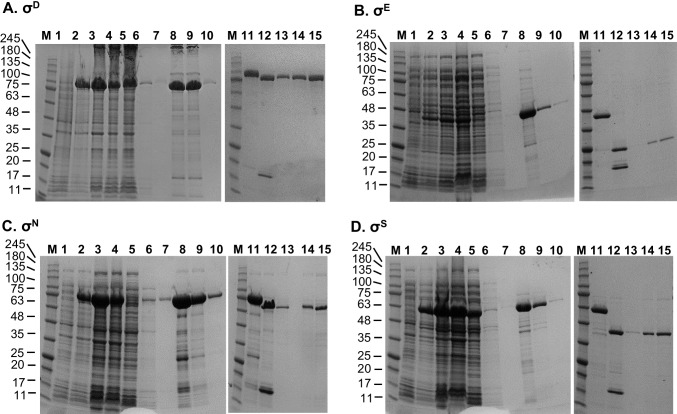
Purification of sigma factors by employing the general purification protocol. **A** σ^D^, **B** σ^E^, **C** σ^N^, and **D** σ^S^, as visualized by Coomassie blue-stained SDS-PAGE (10–20%). M, Perfect Tricolor protein ladder (Eurx); 1, uninduced cells; 2, lysate obtained after 3.5-h IPTG induction; 3, sample obtained after sonication; 4, supernatant after centrifugation step; 5, flow-through upon column loading with supernatant; 6, flow-through after applying first portion of wash-buffer; 7, flow-through after applying second portion of wash-buffer; 8, elution, first fraction; 9, elution, second fraction; 10, elution, third fraction; 11, sample after dialysis; 12, after Ulp1 addition; 13, flow-through after applying sample to the column; 14, 2 μl of the sample concentrated with the Amicon filtration device (10 MWCO); 15, 5 μl of the same sample as in 14. Molecular weights of the marker bands are indicated on the left of each gel

**Fig. 3:**
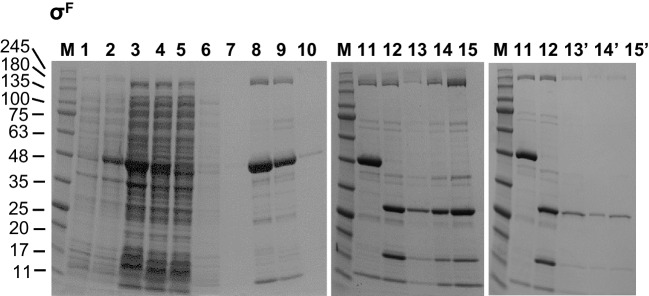
Purification of σ^F^ by employing modified general purification protocol. Lanes marked as M and 1–15 are the same as described in Fig.2. Lane 13’, flow-through upon dilution of sample from lane 15 and another loading on the column; 14’, 2 μl of the sample run in 13’ but concentrated with the Amicon filtration device (10 MWCO); 15’, 5 μl of the same sample as in 14’. See Materials and Methods for details. Molecular weights of the marker bands are indicated on the left of each gel

This procedure worked very well for overexpression and purification of σ^D^, σ^E^, σ^S^, and σ^N^ (Fig. 2). However, in case of σ^F^, σ^H^, and σ^FecI^, some modifications had to be introduced since the preps obtained by the general procedure described above contained substantial amounts of co-purified other proteins (Fig.3 and Fig.S1–2).

### Purification of σ^F^

Here, many protein contaminants were still present after the second column and His_8_-SUMO tag removal (Fig. 3, lanes 13–15). We suspected that some of these contaminants were enriched after column dead-volume wash and thus we applied a rather simple remedy—the final sample was diluted in a large volume (12 ml) of the 1 x TGED buffer, and the whole sample was re-applied onto fresh Ni^2+^-NTA resin. Only the flow-through fraction was this time collected and then concentrated. As shown in Fig. 3 (lanes 13’–15’), most of the high-molecular weight proteins were removed. Although this resulted in σ^F^ losses, the sample purity was enhanced.

### σ^H^ and σ^FecI^ purification

These two sigma factors were the most difficult to purify due to the prep’s high contamination with co-purified proteins when applying the general purification procedure (Fig. S1A and S2).

In case of σ^H^, we suspected that the contaminants were in large part chaperone proteins (such as DnaK, DnaJ, GrpE, and/or GroEL/GroES) that bind σ^H^ under non-heat shock conditions to prevent its activity [Arsène et al. 2000; Guisbert et al. 2004]. Such interactions were reported in other papers dealing with σ^H^ purification [e.g., Liberek et al. 1992]. On the other hand, it was reported that chaperone (DnaK) contamination of a given protein prep could be overcome by adding ATP, Mg^2+^, and denatured proteins to the wash buffer [Rial and Ceccarelli 2002]. These allow for the chaperone unbinding from σ^H^ and binding to their preferred substrates, i.e., the added denatured proteins. In our case, this procedure helped in removing some, but not all of the co-purified proteins (Fig. S1B).

**Fig. 4:**
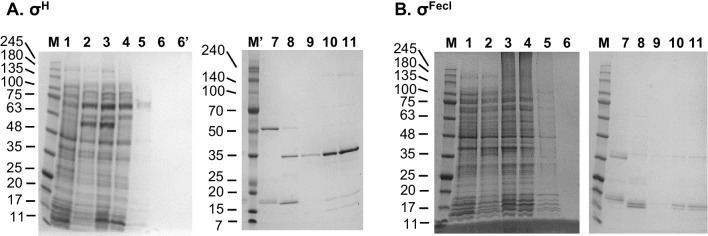
Purification of σ^H^ and σ^FecI^ by employing denaturing conditions. **A** σ^H^, **B** σ^FecI^, as visualized by Coomassie blue-stained SDS-PAGE (10–20%). M and M’, Perfect Tricolor protein ladder (Eurx); 1, uninduced cells; 2, lysate obtained after 3.5-h IPTG induction; 3, sample obtained after sonication and subsequent centrifugation; 4, flow-through; 5, flow-through after applying first portion of wash buffer; 6, flow-through after applying second portion of wash buffer; 6’, flow-through after applying ATP/Mg^2+^/denatured proteins (for σ^H^ only, see Materials and Methods for details); 7, sample after dialysis; 8, after Ulp1 addition; 9, flow-through after applying sample to the column; 10, 5 μl of the sample concentrated with the Amicon filtration device (10 MWCO),; 11, 10 μl of the same sample as in 10. Molecular weights of the marker bands are indicated on the left of each gel

We thus decided to undertake a different approach, where in addition to the steps just described, we applied denaturing conditions to unbind proteins that specifically and tightly interact with σ^H^. Thus, 6M guanidine hydrochloride buffer was used to wash the resin with bound His-tagged proteins. The His_8_-SUMO tagged σ^H^ was then eluted under the same conditions and was brought back to its native state by subsequent dialysis in buffers with decreasing concentrations of another denaturant, i.e. urea. The ensuing steps (His_8_-SUMO tag cleavage and following procedures) were the same as in the general protocol. This way, we finally succeeded in obtaining pure σ^H^ protein preps (Fig. 4A).

In case of σ^FecI^, upon initial purification, we observed contamination with many proteins, among which four were predominant (~20 kDa, ~35 kDa, ~75 kDa, and ~180 kDa, Fig. S2). It is known that σ^FecI^ tightly interacts with FecR (35 kDa) which in turn also interacts with FecA (85 kDa) [Enz et al. 2000]. The apparent molecular weights of two of the co-purified proteins roughly correspond to FecR and FecA, although their identity remains unresolved.

In our first attempt at obtaining pure σ^FecI^, we decided to increase salt concentration of the wash buffer to 1 M as this sometimes helps to remove unwanted interactions (see, e.g., [Sobala et al. 2019]). However, this did not yield any improvement (data not shown). Thus, we undertook the same approach as for σ^H^, i.e., 6M guanidine hydrochloride was used to denature and unbind any potential proteins directly and tightly interacting with σ^FecI^. In this case, the procedure turned out to be more successful, although some proteins still co-purified with this sigma factor (Fig. 4B).

### Protein yields and sample purity

**Fig. 5:**
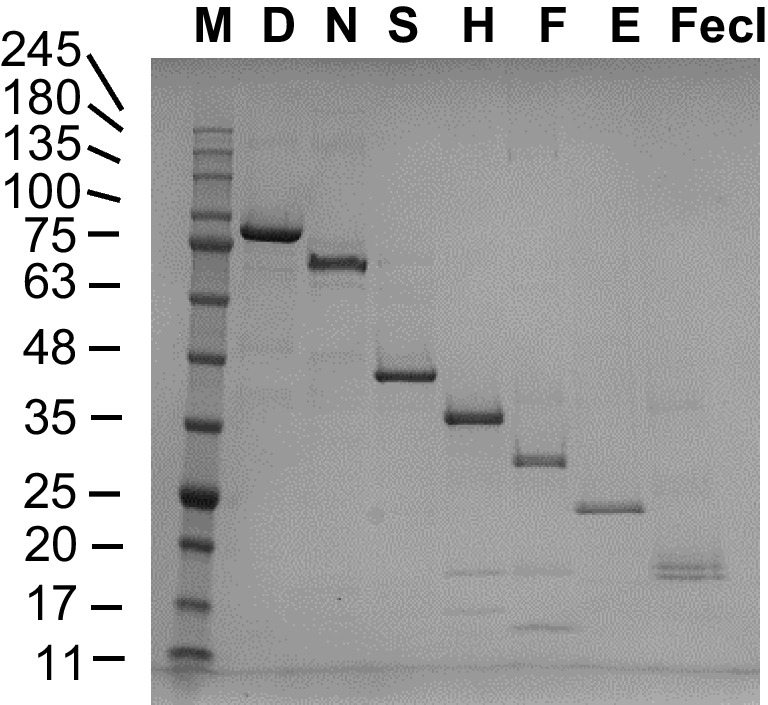
Purified sigma factors visualized by Coomassie blue-stained SDS-PAGE (10–20%). M, Perfect Tricolor protein ladder (Eurx); D, σ^D^ (M.w. ~70 kDa); N, σ^N^ (M.w. ~54 kDa); S, σ^S^ (M.w. ~38 kDa); H, σ^H^ (M.w. ~32 kDa); F, σ^F^ (M.w. ~28 kDa); E, σ^E^ (M.w. ~24 kDa); FecI, σ^FecI^ (M.w. ~19 kDa). About 1.5 μg of each protein were loaded per lane. Molecular weights of the marker bands are indicated on the left of the gel

Protein yields for each sigma factor, calculated per 1 g of cell wet weight, are given in Table 1. To assess sample purity, preps of the final purified proteins were resolved by SDS-PAGE, which was followed by Coomassie blue-staining and densitometry (Fig. 5 and Table 1). It should be noted that it is known that sigma factors migrate differently in SDS-PAGE than it would be expected from their molecular weight [Helmann 2019]. For example, σ^D^ whose molecular weight is 70 kDa migrates around 90 kDa. Similarly, σ^N^ migrates much slower than expected for a 54 kDa protein.

**Table 1:** Examples of protein yields obtained for each sigma factor that was purified in this study. OD_600_ values given correspond to optical density of cell culture at the point that IPTG induced cells were harvested. The IPTG induction was initiated at OD_600_~0.4, and was carried out for ~3.5 h. Purity was assessed by densitometry of Coomassie blue-stained gels

Sigma factor	Total mg of purified protein/250 ml cell culture	OD_600_	Yield (mg protein/1 g wet cell weight)	Purity
σ^D^	1.48	2.56	1.36	98%
σ^E^	0.42	0.64	1.53	96%
σ^F^	0.04	1.70	0.05	82%
σ^H^	0.28	1.69	0.39	98%
σ^N^	0.98	2.08	1.11	95%
σ^S^	0.41	2.23	0.42	93%
σ^FecI^	0.12	1.78	0.15	68%

When taking into account the amount of protein obtained per 250 ml of IPTG induced cell culture, the highest amount was obtained for σ^D^. *E. coli* culture overexpressing that sigma factor also grew the best after IPTG addition. On the other hand, when looking at the overall yield that takes into account cell mass (expressed as mg protein/1 g cell wet weight), it is evident that the highest yield was obtained for σ^E^. Induction of that factor’s overproduction had a significantly detrimental effect on cell growth; however, σ^E^ production had proceeded nonetheless (data not shown). For five sigma factors (σ^D^, σ^E^, σ^H^, σ^N^, σ^S^), sample purity was ≥ 93%, with the highest purity obtained for σ^D^ and σ^H^ (98%).

The least efficient was σ^F^ purification even though its overproduction did not severely impair growth. In order to obtain higher amounts of σ^F^, larger cell culture volumes and/or longer induction times, growing cells at different temperatures, using different IPTG concentrations or inducing at different OD values should be used in the future and efforts taken to increase sample purity, such as employing a gel filtration or ion exchange column, instead of simply reloading sample on the nickel column after SUMO-tag removal. The same is true for σ^FecI^ whose purity was the lowest. Nevertheless, we deemed the obtained protein preps to be much increased in purity in comparison to the general protocol first used and decided to proceed with assessment of the activity of all sigma factor samples.

### Assessment of the purified sigma factor activity

In order to assess whether the purified sigma factors obtained are active, i.e., whether they could form a functional holoenzyme with core RNAP, we employed EMSA assays. Here, each sigma factor was pre-incubated with core RNAP to form the holoenzyme, and then appropriate Cy5-labeled DNA template was added. Promoter regions specifically recognized by each sigma factor were chosen based on literature reports and were as follows: *greAp1-p4* promoter region for σ^D^ and σ^E^ [Potrykus et al. 2010; Dylewski et al. 2019]; p*flgM* for σ^F^ [Park et al. 2001]; p*groE* for σ^H^ [Nonaka et al. 2006; Wade et al. 2006]; p*4relA* for σ^N^ [Brown et al. 2014]; p*xapA* for σ^S^ [Maciag et al. 2011]; and p*fecA* for σ^FecI^ [Enz et al. 2003].

The samples were then loaded onto a running native PAGE, and a shift in the DNA’s electrophoretic migration indicated that a given DNA fragment was bound by proteins in the sample. As control, samples with core RNAP only or sigma factor only were also included. A shift in band migration occurring with the holoenzyme, but not with these factors alone, indicates that the interaction is specific to the whole complex, and thus the sigma factor under investigation is active in recognizing its corresponding promoter region when bound in the holoenzyme.

**Fig. 6:**
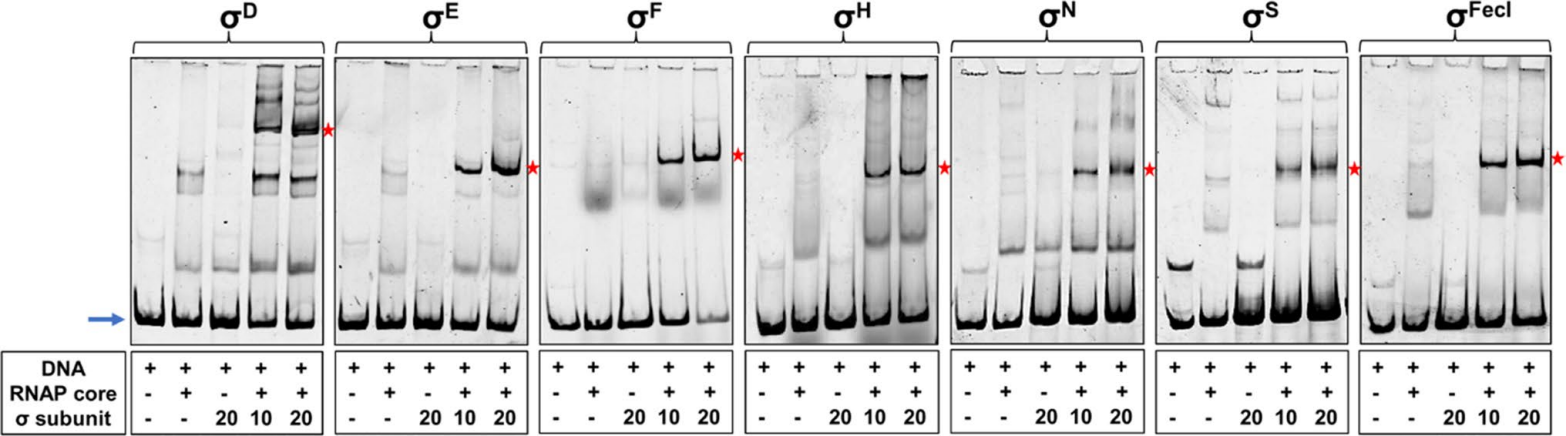
EMSA studies demonstrate that all purified sigma factors are capable of forming functional holoenzyme with core RNAP. Cy5-labeled promoter template was incubated with the *E. coli* core RNAP and/or with 10 or 20 fold molar excess of appropriate sigma factor over core RNAP, as indicated in the figure. Blue arrow and red asterisks denote positions of unbound DNA and major DNA-RNAP holoenzyme complexes, respectively

As can be seen in Fig. 6, all sigma factors purified here were able to form a functional holoenzyme with core RNAP. Band shifts were observed for all σ-core RNAP complexes, even for σ^F^, whose yield was the least efficient and purity was only 82%. In addition, to our knowledge, the p*flgM* promoter region used here for σ^F^ holoenzyme was never mapped experimentally nor in vitro transcription data was reported to date for this promoter; its -10 and -35 regions were only inferred from DNA sequence [Park et al. 2001]. Here, we cannot confirm that these regions were attributed correctly; nevertheless, we do confirm that this region contains a σ^F^-dependent promoter that is recognized by RNAP holoenzyme in vitro and that the σ^F^ obtained is active in promoter recognition when combined with RNAP core enzyme.

Another drawback could have been foreseen for σ^FecI^ whose yield was second to last and the prep was substantially contaminated with co-purified proteins (only 68% purity). As stated above, we speculate that one of those co-purified proteins might be FecR (M.w. 35 kDa). Interestingly, the σ^FecI^ interaction with FecR N-terminal domain was reported to be essential for σ^FecI^ functionality in transcription initiation [Ochs et al. 1995; Mahren et al. 2002]. It could be that fortuitously, the prep obtained might thus indeed contain the necessary σ^FecI^ partner, although not at stoichiometric concentrations, as judged by the Coomassie blue-stained SDS-PAGE gel (Fig. 4B and Fig. 5). However, that requires verification in future studies.

## Concluding remarks

**Fig. 7:**
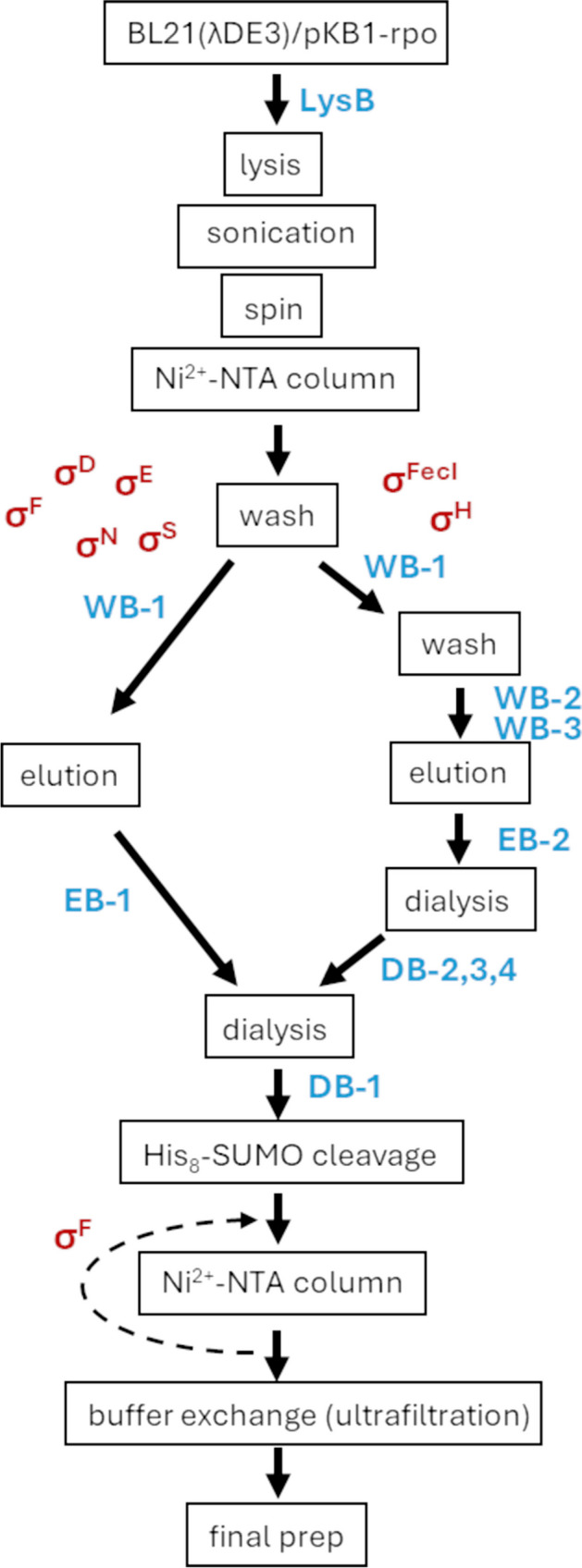
Schematic representation of RNA polymerase sigma factor purification. All sigma factors purified are able to form a functional holoenzyme with core RNAP. Protocol details are provided in the Materials and Methods section. In blue, buffers used; in red, sigma factors purified

Here, we provide a simple and unified protocol for overproduction and purification of all seven *E.coli* RNAP sigma factors. The use of His_8_-SUMO tag had allowed obtaining all of these factors in the soluble form, unlike other protocols where some of these factors had to be extracted from inclusion bodies. We believe that the method presented is also elegant since it employs only one type of resin and after His_8_-SUMO tag removal by treatment with a specific protease (Ulp1) all sigma factors are in their native form. The established purification scheme is summarized in Fig. 7.

The general protocol employed proved successful for purification of four factors (σ^D^, σ^E^, σ^S^, and σ^N^) and upon slight modification for σ^F^ as well. In order to purify σ^H^ and σ^FecI^ away from proteins that tightly bind to them, denaturing conditions had to be applied. Still, all seven sigma factors obtained are active—they are able to form a functional holoenzyme with core RNAP, as we demonstrated by EMSA studies.

With the boom of high throughput techniques, it might seem that in vitro studies with purified factors to study transcription are obsolete. However, this is not the case, as dissecting specific molecular mechanisms at transcriptional level still requires obtaining pure proteins, whether it be for in vitro transcription, single molecule in vitro studies, mapping RNAP binding sites on DNA or establishing crystal structures of RNAP complexes. We thus believe that the protocols for purification of the seven *E. coli* sigma factors that we report here may be helpful to many others and in addition may be adapted for purification of sigma factors from other bacterial species as well.

### Supplementary information

ESM 1 (PDF 873 kb)
