# Supplementary material for: A simple and unified protocol to purify all seven Escherichia coli RNA polymerase sigma factors
Source: J Appl Genet. 2024 May 6;65(3):615–25. doi: 10.1007/s13353-024-00870-3 (PMC11310293; doi:10.1007/s13353-024-00870-3)
Supplement: Supplementary file 1 — (PDF 873 kb) [file 13353_2024_870_MOESM1_ESM.pdf]

Supplementary data for:

A simple and unified protocol to purify all seven *Escherichia coli* RNA polymerase sigma factors

Barbara Kędzierska[1], Aleksandra Stodolna[1], Katarzyna Bryszkowska[1], Maciej Dylewski[1] and Katarzyna Potrykus[1]*

[1] Department of Bacterial Molecular Genetics, Faculty of Biology, University of Gdańsk, Gdańsk, Poland

*for correspondence: Katarzyna Potrykus (ORCID ID 0000-0002-4902-3348), katarzyna.potrykus@ug.edu.pl

Sequence of the *rpoD* synthetic DNA fragment (GeneArt, Thermo Fisher Scientific).
Underlined – *Nde*I site; in bold – *Bpu*1102I site.

TCGAGCATATGGAGCAAAACCCGCAGTCACAGCTGAAACTTCTTGTCACCCGTGGTAAGGAGCAAGGCTATCTGACCTAT
GCCGAGGTCAATGACCATCTGCCGGAAGATATCGTCGATTCAGATCAGATCGAAGACATCATCCAAATGATCAACGACAT
GGGCATTCAGGTGATGGAAGAAGCACCGGATGCCGATGATCTGATGCTGGCTGAAAACACCGCGGACGAAGATGCTGCCG
AAGCCGCCGCGCAGGTGCTTTCCAGCGTGGAATCTGAAATCGGGCGCACGACTGACCCGGTACGCATGTACATGCGTGAA
ATGGGCACCGTTGAACTGTTGACCCGCGAAGGCGAAATTGACATCGCAAAGCGTATTGAAGACGGGATCAACCAGGTTCA
ATGCTCCGTTGCTGAATATCCGGAAGCGATCACCTATCTGCTGGAACAGTACGATCGTGTTGAAGCAGAAGAAGCGCGTC
TGTCCGATCTGATCACCGGCTTTGTTGACCCGAACGCAGAAGAAGATCTGGCACCTACCGCCACTCACGTCGGTTCTGAG
CTTTCCCAGGAAGATCTGGACGATGACGAAGATGAAGACGAAGAAGATGGCGATGACGACAGCGCCGATGATGACAACAG
CATCGACCCGGAACTGGCTCGCGAAAAATTTGCGGAACTACGCGCTCAGTACGTTGTAACGCGTGACACCATCAAAGCGA
AAGGTCGCAGTCACGCTACCGCTCAGGAAGAGATCCTGAAACTGTCTGAAGTATTCAAACAGTTCCGCCTGGTGCCGAAG
CAGTTTGACTACCTGGTCAACAGCATGCGCGTCATGATGGACCGCGTTCGTACGCAAGAACGTCTGATCATGAAGCTCTG
CGTTGAGCAGTGCAAAATGCCGAAGAAAAACTTCATTACCCTGTTTACCGGCAACGAAACCAGCGATACCTGGTTCAACG
CGGCAATTGCGATGAACAAGCCGTGGTCGGAAAAACTGCACGATGTCTCTGAAGAAGTGCATCGCGCCCTGCAAAAACTG
CAGCAGATTGAAGAAGAAACCGGCCTGACCATCGAGCAGGTTAAAGATATCAACCGTCGTATGTCCATCGGTGAAGCGAA
AGCCCGCCGTGCGAAGAAAGAGATGGTTGAAGCGAACTTACGTCTGGTTATTTCTATCGCTAAGAAATACACCAACCGTG
GCTTGCAGTTCCTTGACCTGATTCAGGAAGGCAACATCGGTCTGATGAAAGCGGTTGATAAATTCGAATACCGCCGTGGT
TACAAGTTCTCCACCTACGCAACCTGGTGGATCCGTCAGGCGATCACCCGCTCTATCGCGGATCAGGCGCGCACCATCCG
TATTCCGGTGCACATGATTGAGACCATCAACAAGCTCAACCGTATTTCTCGCCAGATGCTGCAAGAGATGGGCCGTGAAC
CGACGCCGGAAGAACTGGCTGAACGTATGCTGATGCCGGAAGACAAGATCCGCAAAGTGCTGAAGATCGCCAAAGAGCCA
ATCTCCATGGAAACGCCGATCGGTGATGATGAAGATTCGCATCTGGGGGATTTCATCGAGGATACCACCCTCGAGCTGCC
GCTGGATTCTGCGACCACCGAAAGCCTGCGTGCGGCAACGCACGACGTGCTGGCTGGCCTGACCGCGCGTGAAGCAAAAG
TTCTGCGTATGCGTTTCGGTATCGATATGAACACCGACTACACGCTGGAAGAAGTGGGTAAACAGTTCGACGTTACCCGC
GAACGTATCCGTCAGATCGAAGCGAAGGCGCTGCGCAAACTGCGTCACCCGAGCCGTTCTGAAGTGCTGCGTAGCTTCCT
GGACGATTAA**GCTGAGC**AATA

Table S1. Plasmids used in this study

Plasmid	Description	Source
pKB1	pET28a derivative (cloning region:T7 promoter – *lac* operator – *Xba*I site – His8x – SUMO tag – *Nde*I site – gene of interest – *Bpu*1102I site – T7 terminator); kan^{R}	This work
pKB1-rpoD	*rpoD* gene inserted between *Nde*I/*Bpu*1102I sites of pKB1	This work
pKB1-rpoE	*rpoE* gene inserted between *Nde*I/*Bpu*1102I sites of pKB1	This work
pKB1-rpoF	*rpoF* gene inserted between *Nde*I/*Bpu*1102I sites of pKB1	This work
pKB1-rpoH	*rpoH* gene inserted between *Nde*I/*Bpu*1102I sites of pKB1	This work
pKB1-rpoN	*rpoN* gene inserted between *Nde*I/*Bpu*1102I sites of pKB1	This work
pKB1-rpoS	*rpoS* gene inserted between *Nde*I/*Bpu*1102I sites of pKB1	This work
pKB1-fecI	*fecI* gene inserted between *Nde*I/*Bpu*1102I sites of pKB1	This work

Table S2. Primers used for PCR amplification of the sigma subunit genes (underlined – *Nde*I sites; in bold – *Bpu*1102I sites; highlighted in grey – substitutions introducing silent mutations destroying native *Bpu*1102I sites present in the target gene sequence)

Primer	Sequence (5' – 3')	Description
AS12	ATTCGAGCATATGGAGCAAAACCCGC	*rpoD* forward primer
KPr118	TATT**GCTCAGC**TTAATCGTCCAGGAAGCTACGCAG	*rpoD* reverse primer
KPr115	TCGAGCATATGAGCGAGCAGTTAACGGACCAG	*rpoE* forward primer
KPr116	TATT**GCTCAGC**TCAACGCCTGATAAGCGGTTGA	*rpoE* reverse primer
AS13	ATTCGAGCATATGGTGAATTCACTCTATACCGC	*rpoF* forward primer
AS2	TATT**GCTCAGC**TTATAACTTACCCAGTTTAGTGCG	*rpoF* reverse primer
AS14	GCTCGAGCATATGACTGACAAAATGCAAAGTTTAGC	*rpoH* forward primer, fragment 1
AS10	TACACGCTCGGCGGAAAC	*rpoH* reverse primer fragment 1
AS9	GTTTCCGCCGAGCGTGTA	*rpoH* forward primer, fragment 2
AS8	TATT**GCTCAGC**TTACGCTTCAATGGCAG	*rpoH* reverse primer fragment 2
AS15	ATTCGAGCATATGAAGCAAGGTTTGCAACTCAGACTTAGCC	*rpoN* forward primer
AS6	TATT**GCTCAGC**TCAAACGAGTTGTTTACGCTGGTT	*rpoN* reverse primer
AS11	ATTCGAGCATATGAGTCAGAATACGCTGA	*rpoS* forward primer
KPr122	TATT**GCTCAGC**TTACTCGCGGAACAGCGCTT	*rpoS* reverse primer
AS3	TCGAGCATATGTCTGACCGCGC	*fecI* forward primer
AS4	TATT**GCTCAGC**TCATAACCCATACTCCAGAC	*fecI* reverse primer

Table S3. Primers used for PCR amplification of DNA fragments for EMSA studies

Primer	Sequence (5'- 3')	Description	
KPr151	Cy5-CAGGAATTGGGGATCGGAATTC	forward primer	for amplifying p*greA*$_{all}$-*lacZ* promoter region (contains σ^D and σ^E dependent promoters); fragment size: 380 bp
KPr53	GCCAGGGTTT TCCCAGTCAC GACG	reverse primer	
KPr147	Cy5-GTGCGCATGGTATCGGGACA	forward primer	for amplifying p*flgM* promoter region (contains σ^F dependent promoter); fragment size: 201 bp
KPr148	TAAGCACCGTTCAACCGCGC	reverse primer	
KPr183	Cy5-CGATGGTAGCACAATCAGATTCG	forward primer	for amplifying p*groE* promoter region (contains σ^H dependent promoter); fragment size: 333 bp
KPr184	CTGCAGCGGCTAAATCCA	reverse primer	
KPr141	Cy5 - CACAGCAACTTCAGATGGGG	forward primer	for amplifying p4*relA* promoter region (contains σ^N dependent promoter); fragment size: 336 bp
KPr142	CGTTTGAGTCATACCAGGGC	reverse primer	
KPr179	Cy5-TAATGCAAAGGAATTAATATCGCCAA	forward primer	for amplifying p*xapA* promoter region (contains σ^S dependent promoter); fragment size: 255 bp
KPr180	CTTATAAACCTGATTTCACGCCAC	reverse primer	
KPr149	Cy5-CGATCCTGAACGTTATCGC	forward primer	for amplifying p*fecA* promoter region (contains σ^{FecI} dependent promoter); fragment size: 217 bp
KPr150	ACAACACCTTTGGTTAACACC	reverse primer	

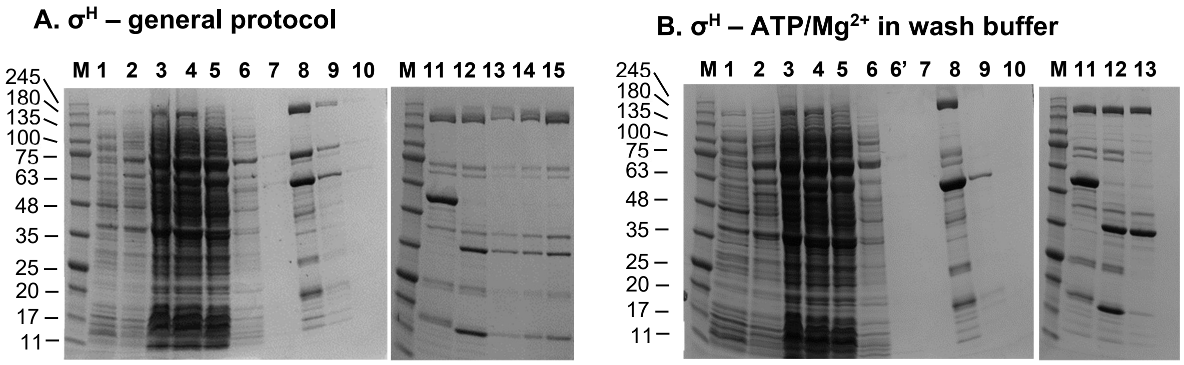

Figure S1. Purification of σ^H by employing the general and modified purification protocols.

A. outcome of employing general protocol, as visualized by Coomassie blue-stained SDS-PAGE (10 – 20%). M – Perfect Tricolor Protein Ladder (Eurx), 1 – uninduced cells, 2 – lysate obtained after 3.5 hr IPTG induction, 3 – sample obtained after sonication, 4 – supernatant after centrifugation step, 5 – flow-through upon column loading with supernatant, 6 - flow-through after applying first portion of wash-buffer, 7 - flow-through after applying second portion of wash-buffer, 8 – elution, first fraction, 9 – elution, second fraction, 10 – elution, third fraction, 11 – sample after dialysis, 12 – after Ulp1 addition, 13 – flow-through after applying sample to the column, 14 – 2 µl of the sample concentrated with the Amicon filtration device (10 MWCO), 15 – 5 µl of the same sample as in 14. Molecular weights of the marker bands are indicated on the left of each gel.

B. outcome of employing modified protocol; lanes M, 1-13 are the same as described for A. In addition, lane 6’ – flow-through after wash with a buffer containing ATP, Mg^{2+} and denatured proteins (see Materials and Methods of the main manuscript for details).

σ^{FecI} – general protocol

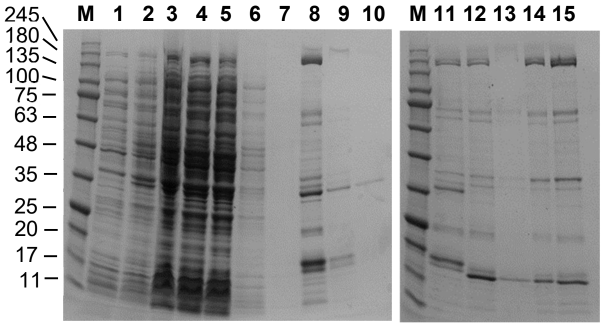

Figure S2. Purification of σ^{FecI} by employing the general purification protocols, as visualized by Coomassie blue-stained SDS-PAGE (10 – 20%). M – Perfect Tricolor Protein Ladder (Eurx), 1 – uninduced cells, 2 – lysate obtained after 3.5 hr IPTG induction, 3 – sample obtained after sonication, 4 – supernatant after centrifugation step, 5 – flow-through upon column loading with supernatant, 6 - flow-through after applying first portion of wash-buffer, 7 - flow-through after applying second portion of wash-buffer, 8 – elution, first fraction, 9 – elution, second fraction, 10 – elution, third fraction, 11 – sample after dialysis, 12 – after Ulp1 addition, 13 – flow-through after applying sample to the column, 14 – 2 µl of the sample concentrated with the Amicon filtration device (10 MWCO), 15 – 5 µl of the same sample as in 14. Molecular weights of the marker bands are indicated on the left of each gel.
